# The Pulse in Peritonitis

**Published:** 1901-05-25

**Authors:** 


### THE PULSE IN PERITONITIS.

Again and again it has been pointed out that the pulse is the great guide when the peritoneum is involved, and that, notwithstanding the favourable character of other symptoms, if the pulse remains quick we have no right to be satisfied with the condition. The converse, however, may not hold true. A case recorded by Bowlby and Steedman[1] serves well to show how careful we must be not to place reliance upon single symptoms; for here, although the abdominal cavity contained about a quart of darkly blood-stained fluid, and the greater part of the small intestine was so twisted as to form a large volvulus, the pulse remained below 100 in spite of the strangulation of so large a mass of intestine, and although the patient was evidently much collapsed and in intense pain. The case was a very remarkable one in other respects, this volvulus having come on after a previous operation for perforated gastric ulcer in which rigors had occurred; but the authors draw special attention to this question of pulse-rate, saying: "It is seldom that so serious a condition as was found in this case on opening the abdomen causes so little disturbance of the pulse-rate; but this case, and others which we have seen, should serve as a warning not to place too much reliance on a quiet pulse when the question of laparotomy is under consideration for the relief of such suspected conditions as perforated gastric ulcer and volvulus."

[1] Lancet, May. 4.

